# Analyzing ChIP-chip Data Using Bioconductor

**DOI:** 10.1371/journal.pcbi.1000227

**Published:** 2008-11-28

**Authors:** Joern Toedling, Wolfgang Huber

**Affiliations:** EMBL European Bioinformatics Institute, Wellcome Trust Genome Campus, Hinxton, United Kingdom; Whitehead Institute, United States of America

## Introduction

ChIP-chip, chromatin immunoprecipitation combined with DNA microarrays, is a widely used assay for DNA–protein binding and chromatin plasticity, which are of fundamental interest for the understanding of gene regulation.

The interpretation of ChIP-chip data poses two computational challenges: first, what can be termed primary statistical analysis, which includes quality assessment, data normalization and transformation, and the calling of regions of interest; second, integrative bioinformatic analysis, which interprets the data in the context of existing genome annotation and of related experimental results obtained, for example, from other ChIP-chip or (m)RNA abundance microarray experiments.

Both tasks rely heavily on visualization, which helps to explore the data as well as to present the analysis results. For the primary statistical analysis, some standardization is possible and desirable: commonly used experimental designs and microarray platforms allow the development of relatively standard workflows and statistical procedures. Most software available for ChIP-chip data analysis can be employed in such standardized approaches [1]–[6]. Yet even for primary analysis steps, it may be beneficial to adapt them to specific experiments, and hence it is desirable that software offers flexibility in the choice of algorithms for normalization, visualization, and identification of enriched regions.

For the second task, integrative bioinformatic analysis, the datasets, questions, and applicable methods are diverse, and a degree of flexibility is needed that often can only be achieved in a programmable environment. In such an environment, users are not limited to predefined functions, such as the ones made available as “buttons” in a GUI, but can supply custom functions that are designed toward the analysis at hand.

Bioconductor [7] is an open source and open development software project for the analysis and comprehension of genomic data, and it offers tools that cover a broad range of computational methods, visualizations, and experimental data types, and is designed to allow the construction of scalable, reproducible, and interoperable workflows. A consequence of the wide range of functionality of Bioconductor and its concurrency with research progress in biology and computational statistics is that using its tools can be daunting for a new user. Various books provide a good general introduction to R and Bioconductor (e.g., [8]–[10]), and most Bioconductor packages are accompanied by extensive documentation. This tutorial covers basic ChIP-chip data analysis with Bioconductor. Among the packages used are *Ringo* [5], *biomaRt* [11], and *topGO* [12].

We wrote this document in the Sweave [13] format, which combines explanatory text and the actual R source code used in this analysis [14]. Thus, the analysis can be reproduced by the reader. An R package *ccTutorial* that contains the data, the text, and code presented here, and supplementary text and code, is available from the Bioconductor Web site.

> *library(“Ringo”*)

> *library(“biomaRt”*)

> *library(“topGO”*)

> *library(“ccTutorial”*)

**Terminology.** *Reporters* are the DNA sequences fixed to the microarray; they are designed to specifically hybridize with corresponding genomic fragments from the immunoprecipitate. A reporter has a unique identifier and a unique sequence, and it can appear in one or multiple *features* on the array surface [15]. The *sample* is the aliquot of immunoprecipitated or *input* DNA that is hybridized to the microarray. We shall call a genomic region apparently enriched by ChIP a *ChIP-enriched region*.

**The data.** We consider a ChIP-chip dataset on a post-translational modification of histone protein H3, namely tri-methylation of its Lysine residue 4, in short H3K4me3. H3K4me3 has been associated with active transcription (e.g., [16],[17]). Here, enrichment for H3K4me3 was investigated in *Mus musculus* brain and heart cells. The microarray platform is a set of four arrays manufactured by NimbleGen containing 390 k reporters each. The reporters were designed to tile 32,482 selected regions of the *Mus musculus* genome (assembly mm5) with one base every 100 bp, with a different set of promoters represented on each of the four arrays ([18], Methods: Condensed array ChIP-chip). We obtained the data from the GEO repository [19] (accession GSE7688).

## Importing the Data into R

For each microarray, the scanner output consists of two files, one holding the Cy3 intensities (the untreated *input* sample), the other one the Cy5 intensities, coming from the immunoprecipitated sample. These files are tab-delimited text files in NimbleGen's *pair* format. Since the reporters are distributed over four arrays, we have 16 files (4 microarrays×2 dyes×2 tissues).

>* pairDir *<*- system.file(“PairData”,package = “ccTutorial”*)

>* list.files(pairDir, pattern = “pair$”)*

[1] “47101_532.pair” “47101_635.pair” “48153_532.pair” “48153_635.pair”

[5] “48158_532.pair” “48158_635.pair” “48170_532.pair” “48170_635.pair”

[9] “48175_532.pair” “48175_635.pair” “48180_532.pair” “48180_635.pair”

[13] “48182_532.pair” “48182_635.pair” “49728_532.pair” “49728_635.pair”

One text file per array describes the samples, including which two *pair* files belong to which sample. Another file, spottypes.text, describes the reporter categories on the arrays.

We read in the raw reporter intensities and obtain four objects of class *RGList*, a class defined in package *limma* [20], one object per array type.

>* RGs *<*- lapply(sprintf(“files_array%d.txt”,1:4*),

+ *readNimblegen, “spottypes.txt”, path = pairDir)*

See Text S1 for an extended description of the data import.

## Quality Assessment

In this step, we check the arrays for obvious artifacts and inconsistencies between array subsets.

First, we look at the spatial distribution of the intensities on each array. See Text S1 for the figure and the source code. We do not see any artifacts such as scratches, bright spots, or scanning-induced patterns that would render parts of the readouts useless.

On all arrays in our set, the Cy3 channel holds the intensities from the untreated *input* sample, and the Cy5 channel holds the immunoprecipitate from brain and heart, respectively. This experiment setup is reflected in the reporter intensity correlation per channel (see Text S1). The correlation between the intensities of the *input* samples is higher than between the ChIP samples (0.877 versus 0.734).

The Bioconductor package *arrayQualityMetrics* offers an extensive set of visualizations and metrics for assessing microarray data quality. Applied to this dataset, *arrayQualityMetrics* also indicates that the data are of good quality.

## Mapping Reporters to the Genome

A mapping of reporters to genomic coordinates is usually provided by the array manufacturer. Often, however, remapping the reporter sequences to the genome may be required. Here, the microarray had been designed on an outdated assembly of the mouse genome (mm5, May 2004). We remapped the reporter sequences to the current assembly (mm9, July 2007).

We used *Exonerate* [21] for the remapping, requiring 97% sequence similarity for a match. See Text S1 for details and the scripts used.

In *Ringo*, the mapping of reporters to the genome is stored in a *probeAnno* class object. Text S1 contains details on its construction.

> data(*“probeAnno”*)

> *allChrs *<*- chromosomeNames(probeAnno)*

## Genome Annotation

We want to relate ChIP-enriched regions to annotated genome elements, such as potential regulatory regions and transcripts. Using the Bioconductor package *biomaRt* [11], we obtain an up-to-date annotation of the mouse genome from the Ensembl database [22].

The source code for creating the annotation table mm9genes is given in Text S1. The table holds the coordinates, Ensembl gene identifiers, MGI symbols, and description of all genes annotated for the *mm9* mouse assembly.

> data(*“mm9genes”*)

> *mm9genes[sample(nrow(mm9genes), 4),*

+ c(*“name”, “chr”, “strand”, “start”, “end”, “symbol”*)]

See Table 1.

**Table 1:** An excerpt of object ‘mm9genes’.

Name	Chr	Strand	Start	End	Symbol
ENSMUSG00000057903	14	1	51044196	51045125	Olfr739
ENSMUSG00000039615	17	−1	25967581	25970306	Stub1
ENSMUSG00000068823	3	1	102824530	102862108	Csde1
ENSMUSG00000006241	9	1	21731915	21740316	2510048L02Rik

Moreover, we used *biomaRt* to retrieve the Gene Ontology (GO) [23] annotation for all genes in the table. Find the source code and further details in Text S1.

> data(*“mm9.gene2GO”*)

For all genes, we stored which reporters, if any, are mapped inside the gene or in its 5 kb upstream region.

> data(*“mm9.g2p”*)

For later use, we determine which genes have a sufficient number—arbitrarily we say five—of reporters mapped to their upstream region or inside and which genes also have one or more GO terms annotated to them.

> *arrayGenes*<*- names(mm9.g2p) [listLen(mm9.g2p)> = 5]*

>* arrayGenesWithGO *<*- intersect(arrayGenes, names(mm9.gene2GO))*

## Preprocessing

For each sample, we compute the log ratios log_2_(Cy5/Cy3) for all reporters. To adjust for systematic dye and labeling biases, we compute Tukey's biweight mean across each sample's log_2_ ratios and subtract it from the individual log_2_ ratios. Each of the four microarray types contains a unique set of reporters. Thus, we preprocess the arrays separately by type and combine the results into one object holding the preprocessed readouts for all reporters.

> *MAs *<*- lapply(RGs, function(thisRG)*

+* preprocess(thisRG[thisRG$genes$Status =  = “Probe”,],*

+*  method = “nimblegen”, returnMAList = TRUE))*

> *MA *<*- do.call(rbind, MAs)*

> *X *<*- asExprSet(MA)*

> *sampleNames(X) *<*- paste(X$Cy5, X$Tissue, sep = “.”*)

The result is an object of class *ExpressionSet*, the Bioconductor class for storing preprocessed microarray data. Note that first creating a *MAList* for each array type, combining them with rbind, and then converting the result into an *ExpressionSet* is only necessary if the reporters are distributed over more than one microarray type. For data of one microarray type only, you can call preprocess with argument ‘returnMAList = FALSE’ and directly obtain the result as an *ExpressionSet*.

The above procedure is the standard method suggested by NimbleGen for ChIP-chip. The appropriate choice of normalization method generally depends on the data at hand, and the need for normalization is inversely related to the quality of the data. *Ringo* and Bioconductor offer many alternative and more sophisticated normalization methods, e.g., using the genomic DNA hybridization as reference [24]. However, due to the smaller dynamic range of the data in the *input* channel, such additional effort seems less worthwhile than, say, for transcription microarrays.

## Visualizing Intensities along the Chromosome

We visualize the preprocessed H3K4me3 ChIP-chip reporter levels around the start of the *Actc1* gene, which encodes the cardiac actin protein.

> *chipAlongChrom(X, chrom = “2”, xlim = c(113.8725e6,113.8835e6), ylim = c(−3,5),*

+* probeAnno = probeAnno, gff = mm9genes, paletteName = ‘Set2’*)

**Figure 1:**
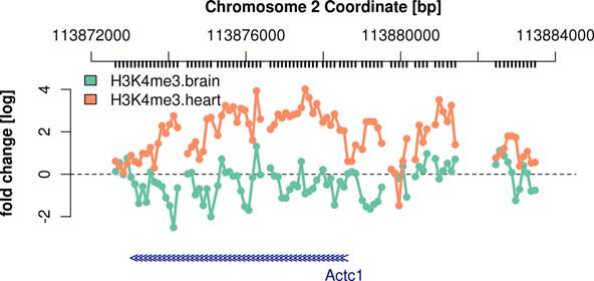
Normalized reporter intensities for H3K4me3 ChIP around the TSS of the *Actc1* gene in *M. musculus* brain and heart cells. The ticks below the genomic coordinate axis on top indicate genomic positions matched by reporters on the microarray. The blue arrows on the bottom mark the *Actc1* gene, with the arrow direction indicating that the gene is located on the Crick strand.

The degree of H3K4me3 enrichment over the reporters mapped to this region seems stronger in heart cells than in brain cells (see Figure 1). However, the signal is highly variable, and individual reporters give different readouts from reporters matching genomic positions only 100 bp away, even though the DNA fragments after sonication are hundreds of base pairs long.

See Text S1 for the corresponding intensities around the start of the brain-specific gene *Crpm1* [25].

When multiple replicates are available, it is instructive to compare these visualizations to assess the agreement between replicates.

## Smoothing of Reporter Intensities

The signal variance arises from systematic and stochastic noise. Individual reporters measure the same amount of DNA with different efficiency due to reporter sequence characteristics [26], such as GC content, secondary structure, and cross-hybridization. To ameliorate these reporter effects as well as the stochastic noise, we perform a smoothing over of individual reporter intensities before looking for ChIP-enriched regions. We slide a window of 900 bp width along the chromosome and replace the intensity at genomic position *x*_0_ by the median over the intensities of those reporters mapped inside the window centered at *x*_0_. Factors to take into account when choosing the width of the sliding window are the size distribution of DNA fragments after sonication and the spacing between reporter matches on the genome.

> *smoothX *<*- computeRunningMedians(X, probeAnno = probeAnno,*

+* modColumn = “Tissue”, allChr = allChrs, winHalfSize = 450, min.probes = 5)*

> *sampleNames(smoothX) *<*- paste(sampleNames(X), “smoothed”,sep = “.”*)

Compare the smoothed reporter intensities with the original ones around the start of the gene *Actc1*.

> *chipAlongChrom(X, chrom = “2”, xlim = c(113.8725e6,113.8835e6), ylim = c(−3,5),*

+* probeAnno = probeAnno, gff = mm9genes, paletteName = ‘Set2’*)

> *chipAlongChrom(smoothX, chrom = “2”, xlim = c(113.8725e6,113.8835e6), ilwd = 4,*

+* probeAnno = probeAnno, paletteName = ‘Dark2’, add = TRUE)*

**Figure 2:**
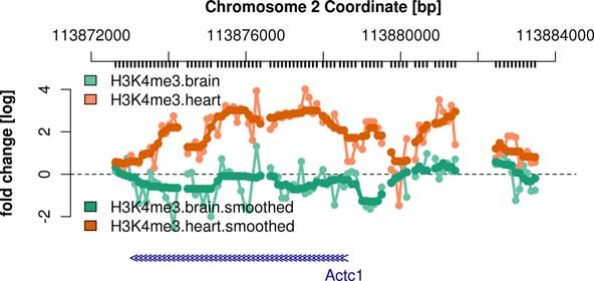
Normalized and smoothed reporter intensities for H3K4me3 ChIP around the TSS of the *Actc1* gene in *M. musculus* brain and heart cells.

See the result in Figure 2. After smoothing, the reporters give a more concise picture that there is H3K4me3 enrichment inside and upstream of *Actc1* in heart but not in brain cells.

## Finding ChIP-Enriched Regions

We would like to determine a discrete set of regions that appear antibody-enriched, together with a quantitative score of our confidence in that and a measure of their enrichment strength. Which approach is best for this purpose depends on the microarray design, on the biological question, and on the subsequent use of the regions, e.g., in a follow-up experiment or computational analysis. Below, we describe one possible approach, but, before that, we discuss two more conceptual aspects.

In the literature, a computed confidence score is often mixed up with the term “*p*-value”. Speaking of a *p*-value is meaningful only if there is a defined null hypothesis and a probability interpretation; these complications are not necessary if the goal is simply to find and rank regions in some way that can be reasonably calibrated.

Furthermore, it is helpful to distinguish between our confidence in an enrichment being present, and the strength of the enrichment. Although stronger enrichments tend to result in stronger signals and hence less ambiguous calls, our certainty about an enrichment should also be affected by reporter coverage, sequence, cross-hybridization, etc.

Let us now consider the following simple approach: for an enriched region, require that the smoothed reporter levels all exceed a certain threshold *y*_0_, that the region contains at least *n*_min_ reporter match positions, and that each of these is less than *d*_max_ basepairs apart from the nearest other affected position in the region.

The minimum number of reporters rule (*n*_min_) might seem redundant with the smoothing median computation (since a smoothed reporter intensity is already the median of all the reporter intensities in the window), but it plays its role in reporter sparse regions, where a window may only contain one or a few reporters. One wants to avoid making calls supported by only few reporters.

The *d*_max_ rule prevents us from calling disconnected regions.

### 

#### Setting the enrichment threshold

The optimal approach for setting the enrichment threshold *y*_0_ would be to tune it by considering sets of positive and negative control regions. As such control regions are often not available, as with the current data, we choose a mixture modeling approach.

The distribution of the smoothed reporter levels *y* can be modeled as a mixture of two underlying distributions. One is the null distribution 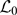 of reporter levels in non-enriched regions; the other is the alternative distribution 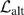 of the levels in enriched regions.

**Figure 3:**
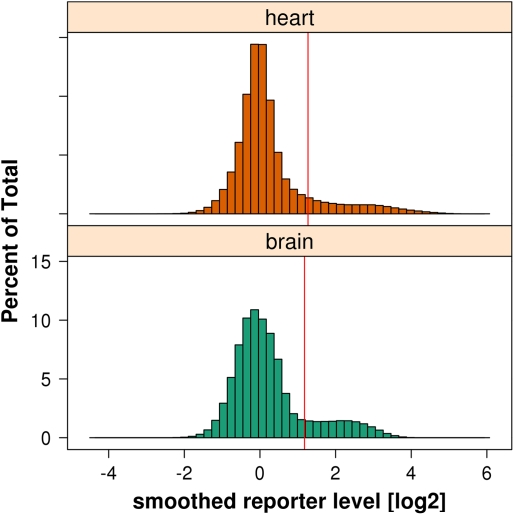
Histograms of reporter intensities after smoothing of reporter levels, measured in *M. musculus* heart and brain cells. The red vertical lines are the cutoff values suggested by the algorithm described in the text.

The challenge is to estimate the null distribution 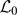. In *Ringo*, an estimate 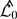 is derived based on the empirical distribution of smoothed reporter levels, as visualized in Figure 3.

>* myPanelHistogram *<*- function(x,…*){

+* panel.histogram(x, col = brewer.pal(8,“Dark2”)[panel.number()],…*)

*+ panel.abline(v = y0[panel.number()], col = “red”*)

+* }*

>* h  = histogram(^∼^y | z,*

+* data = data.frame(*

+*  y = as.vector(exprs(smoothX)),*

+*  z = rep(X$Tissue,each = nrow(smoothX))),*

+* layout =  c(1,2),nint =  50,*

+* xlab =  “smoothed reporter level [log2]”,*

+* panel = myPanelHistogram)*

> *print(h)*

The histograms motivate the following assumptions on the two mixture components 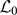 and 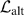: the null distribution 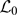 has most of its mass close to its mode *m*_0_, which is close to *y* = 0, and it is symmetric about *m*_0_; the alternative distribution 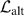 is more spread out and has almost all of its mass to the right of *m*_0_.

Based on these assumptions, we can estimate 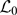 as follows. The mode *m*_0_ can be found by the midpoint of the shorth of those *y* that fall into the interval [−1,1] (on a log_2_scale). The distribution 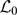 is then estimated from the empirical distribution of *m*_0_−|*y*−*m*_0_|, i.e., by reflecting *y*<*m*_0_ onto *y*>*m*_0_. From the estimated null distribution, an enrichment threshold *y*_0_ can be determined, for example the 99.9% quantile.

>* y0 *<*- apply(exprs(smoothX), 2, upperBoundNull, prob = 0.99)*

The values *y*_0_ estimated in this way are indicated by red vertical lines in the histograms in Figure 3. Antibodies vary in their efficiency to bind to their target epitope, and the noise level in the data depends on the sample DNA. Thus, *y*_0_ should be computed separately for each antibody and cell type, as the null and alternative distributions, 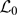 and 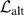, may vary.

This algorithm has been used in previous studies [27]. A critical parameter in algorithms for the detection of ChIP-enriched regions is the fraction of reporters on the array that are expected to show enrichment. For the detection of in-vivo TF binding sites, it is reasonable to assume that this fraction is small, and most published algorithms rely on this assumption. However, the assumption does not necessarily hold for ChIP against histone modifications. The algorithm presented works as long as there is a discernible population of non-enriched reporter levels, even if the fraction of enriched levels is quite large.

Another aspect of ChIP-chip data is the serial correlation between reporters, and there are approaches that aim to model such correlations [28],[29].

#### ChIP-enriched regions

We are now ready to identify H3K4me3 ChIP-enriched regions in the data. We set *n*_min_ = 5 and *d*_max_ = 450.

> *chersX *<*- findChersOnSmoothed(smoothX,*

+* probeAnno =  probeAnno,*

+* thresholds =  y0,*

+* allChr =  allChrs,*

+* distCutOff =  450,*

+* minProbesInRow =  5,*

+* cellType = X$Tissue)*

We relate found ChIP-enriched regions to gene coordinates retrieved from the Ensembl database (see above). An enriched region is regarded as *related* to a gene if its center position is located less than 5 kb upstream of a gene's start coordinate or between a gene's start and end coordinates.

> *chersX *<*- relateChers(chersX, mm9genes, upstream = 5000)*

One characteristic of enriched regions that can be used for ranking them is the *area under the curve* score, that is, the sum of the smoothed reporter levels, each minus the threshold. Alternatively, one can rank them by the highest smoothed reporter level in the enriched region.

> *chersXD *<*- as.data.frame(chersX)*

> *head(chersXD[*

+* order(chersXD$maxLevel, decreasing = TRUE),*

+* c(“chr”, “start”, “end”, “cellType”, “features”, “maxLevel”, “score”)]*)

See Table 2.

**Table 2:** The six ChIP-enriched regions with the highest smoothed reporter levels.

Chr	Start	End	Cell Type	Features	Max. Level	Score
X	7338726	7343630	Heart	ENSMUSG00000000134	5.56	83.6
X	98834348	98838572	Heart	ENSMUSG00000034160	5.45	93.1
17	10508374	10511376	Heart	ENSMUSG00000062078	5.44	76.3
X	148236854	148239554	Heart	ENSMUSG00000025261	5.40	80.3
15	10414592	10416734	Heart	ENSMUSG00000022248 ENSMUSG00000022247	5.39	53.2
17	35972156	35975830	Heart	ENSMUSG00000061607 ENSMUSG00000001525	5.37	62.1

We visualize the intensities around the region with the highest smoothed level.

> *plot(chersX[[which.max(chersXD$maxLevel)]], smoothX, probeAnno = probeAnno,*

+* gff = mm9genes, paletteName = “Dark2”, ylim = c(−1,6))*

**Figure 4:**
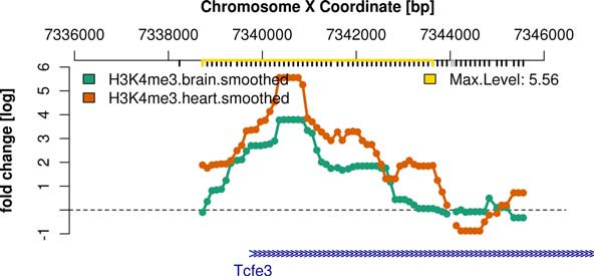
This genomic region is the H3K4me3 ChIP-enriched region with the highest smoothed reporter level.

Figure 4 displays this region, which covers the gene *Tcfe3*.

### Comparing ChIP-Enrichment between the Tissues

There are several ways to compare the H3K4me3 enrichment between the two tissues. How many ChIP-enriched regions do we find in each tissue?

> *table(chersXD$cellType)*

brain heart

11852 10391

Brain cells show a higher number of H3K4me3-enriched regions than heart cells. Which genes show tissue-specific association to H3K4me3 ChIP-enriched regions?

> *brainGenes *<*- getFeats(chersX[sapply(chersX, cellType) =  = “brain”]*)

> *heartGenes *<*- getFeats(chersX[sapply(chersX, cellType) =  = “heart”]*)

> *brainOnlyGenes *<*- setdiff(brainGenes, heartGenes)*

> *heartOnlyGenes *<*- setdiff(heartGenes, brainGenes)*

We use the Bioconductor package *topGO* [12] to investigate whether tissue-specific H3K4me3-enriched genes can be summarized by certain biological themes. *topGO* employs the Fisher test to assess whether among a list of genes, the fraction annotated with a certain GO term is significantly higher than expected by chance, considering all genes that are represented on the microarrays and have GO annotation. We set a *p*-value cutoff of 0.001, and the evaluation starts from the most specific GO nodes in a bottom-up approach. Genes that are used for evaluating a node are not used for evaluating any of its ancestor nodes [12]*elim* algorithm.

> *sigGOTable *<*- function(selGenes, GOgenes = arrayGenesWithGO,*

+* gene2GO = mm9.gene2GO[arrayGenesWithGO], ontology = “BP”,maxP = 0.001)*

+* {*

+* inGenes *<*- factor(as.integer(GOgenes%in%selGenes))*

+* names(inGenes) *<*- GOgenes*

+* GOdata *<*- new(“topGOdata”, ontology = ontology, allGenes = inGenes,*

+*  annot = annFUN.gene2GO, gene2GO = gene2GO)*

+* myTestStat *<*- new(“elimCount”, testStatistic = GOFisherTest,*

+*  name = “Fishertest”, cutOff = maxP)*

+* mySigGroups *<*- getSigGroups(GOdata, myTestStat)*

+* sTab *<*- GenTable(GOdata, mySigGroups, topNodes = length(usedGO(GOdata)))*

+* names(sTab)[length(sTab)] *<*- “p.value”*

+* sTab *<*- subset(sTab, as.numeric(p.value) *<* maxP)*

+* sTab$Term *<*- sapply(mget(sTab$GO.ID, env = GOTERM), Term)*

+* return(sTab)*

+* }*

> *brainRes *<*- sigGOTable(brainOnlyGenes)*

> *print(brainRes)*

See the result GO terms in Table 3. We perform the same analysis for genes showing heart-specific relation to H3K4me3 enrichment.

**Table 3:** GO terms that are significantly over-represented among genes showing H3K4me3 enrichment specifically in brain cells.

GO ID	Term	Annotated	Significant	Expected	*p*-Value
GO:0007268	Synaptic transmission	137	44	24.75	4.1e-05
GO:0007610	Behavior	180	54	32.52	4.9e-05
GO:0007409	Axonogenesis	119	38	21.50	0.00016
GO:0006887	Exocytosis	40	17	7.23	0.00027
GO:0007420	Brain development	136	40	24.57	0.00072

> *heartRes *<*- sigGOTable(heartOnlyGenes)*

> *print(heartRes)*

See the result in Table 4. Genes that show H3K4me3 in brain but not in heart cells are significantly often involved in neuron-specific biological processes. Genes marked by H3K4me3 specifically in heart cells show known cardiomyocyte functions, amongst others.

**Table 4:** GO terms that are significantly over-represented among genes showing H3K4me3 enrichment specifically in heart cells.

GO ID	Term	Annotated	Significant	Expected	*p*-Value
GO:0006936	Muscle contraction	56	13	2.97	4.7e-06
GO:0002526	Acute inflammatory response	17	6	0.90	0.00016
GO:0009887	Organ morphogenesis	339	34	17.95	0.00019
GO:0008016	Regulation of heart contraction	32	8	1.69	0.00019
GO:0030878	Thyroid gland development	7	4	0.37	0.00024
GO:0007512	Adult heart development	8	4	0.42	0.00046
GO:0055003	Cardiac myofibril assembly	4	3	0.21	0.00057
GO:0007507	Heart development	148	21	7.84	0.00090

This analysis could be repeated for the *cellular component* and *molecular function* ontologies of the GO. Besides GO, other databases that collect gene lists can be used for this kind of gene set enrichment analysis. For, example, the Kyoto Encyclopedia of Genes and Genomes (KEGG) [30] is also available in Bioconductor.

In Text S1, we present an additional way for comparing H3K4me3 enrichment between the two tissues, an enriched-region–wise comparison considering the actual overlap of the enriched regions.

### ChIP Results and Expression Microarray Data

We compare the H3K4me3 ChIP-chip results with the expression microarray data, which Barrera et al. [18] provide for the same *M. musculus* tissues they analyzed with ChIP-chip.

> data(*“barreraExpressionX”*)

The data were generated using the Mouse_430_2 oligonucleotide microarray platform from Affymetrix and preprocessed using Affymetrix's MAS5 method. Using *biomaRt*, we created a mapping of Ensembl gene identifiers to the probe set identifiers on that microarray platform (see Text S1 for the source code).

> data(*“arrayGenesToProbeSets”*)

We obtain the expression values for genes related to H3K4me3-enriched regions in heart or brain cells.

> *bX *<*- exprs(barreraExpressionX)*

> *allH3K4me3Genes *<*- union(brainGenes, heartGenes)*

> *allH3K4ProbeSets *<*- unlist(arrayGenesToProbeSets[allH3K4me3Genes]*)

> *noH3K4ProbeSets *<*- setdiff(rownames(bX), allH3K4ProbeSets)*

> *brainH3K4ExclProbeSets *<*- unlist(arrayGenesToProbeSets[brainOnlyGenes]*)

> *heartH3K4ExclProbeSets *<*- unlist(arrayGenesToProbeSets[heartOnlyGenes]*)

> *brainIdx *<*- barreraExpressionX$Tissue =  = “Brain”*

> *brainExpression *<*- list(*

+* H3K4me3BrainNoHeartNo =  bX[noH3K4ProbeSets, brainIdx],*

+* H3K4me3BrainYes =  bX[allH3K4ProbeSets, brainIdx],*

+* H3K4me3BrainYesHeartNo =  bX[brainH3K4ExclProbeSets, brainIdx],*

+* H3K4me3BrainNoHeartYes =  bX[heartH3K4ExclProbeSets, brainIdx]*

+* )*

We use boxplots to compare the brain expression levels of genes with and without H3K4me3-enriched regions in brain/heart cells.

> *boxplot(brainExpression, col = c(“#666666”, “#999966”, “#669966”, “#996666”*),

+* names = NA, varwidth = TRUE, log = “y”,*

+* ylab = ‘geneexpressionlevelinbraincells’*)

> *mtext(side = 1, at = 1:length(brainExpression), padj = 1, font = 2,*

+* text = rep(“H3K4me3”,4), line = 1)*

> *mtext(side = 1, at = c(0.2,1:length(brainExpression)), padj = 1, font = 2,*

+* text = c(“brain/heart”, “−/−”, “+/+”, “+/−”, “−/+”), line = 2)*

**Figure 5:**
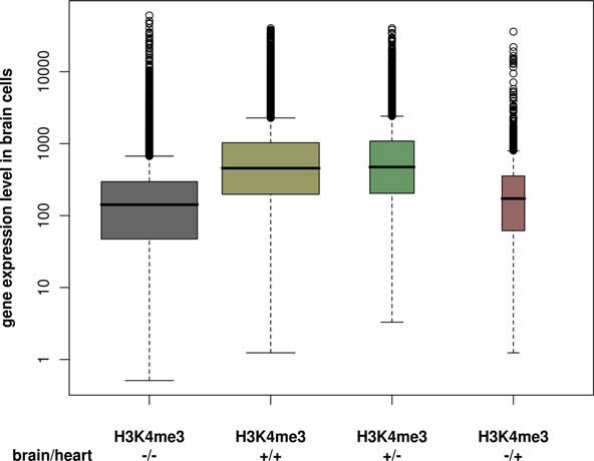
Boxplots for comparing gene expression levels in brain cells. Genes are stratified by whether or not they are related to H3K4me3 ChIP-enriched regions in brain and/or heart cells according to ChIP-chip. The width of the boxes is proportional to the number of genes in each stratification group.

See the boxplots in Figure 5. Genes related to H3K4me3 ChIP-enriched regions show higher expression levels than those that are not, as we can assess using the Wilcoxon rank sum test.

*> with(brainExpression,*

*+ wilcox.test(H3K4me3BrainYesHeartNo, H3K4me3BrainNoHeartNo,*

*+  alternative = “greater”))*

Wilcoxon rank sum test with continuity correction

data: H3K4me3BrainYesHeartNoandH3K4me3BrainNoHeartNo

W  =  88159233, p-value < 2.2e-16

alternative hypothesis: true location shift is greater than 0

### Discussion

The analysis of the ChIP-chip and transcription data of Barrera et al. [18] showed that genes that are expressed in specific tissues are marked by tissue-specific H3K4me3 modification. This finding agrees with previous reports that H3K4me3 is a marker of active gene transcription [16].

We have shown how to use the freely available tools R and Bioconductor for the analysis of ChIP-chip data. We demonstrated ways to assess data quality, to visualize the data, and to find ChIP-enriched regions.

As with any high-throughput technology, there are aspects of ChIP-chip experiments that need close attention, such as specificity and sensitivity of the antibodies, and potential cross-hybridization of the microarray reporters. Good experiments will contain appropriate controls, in the presence of which the software can be used to monitor and assess these issues.

In addition to the ones introduced here, there are other Bioconductor packages that provide further functionality, e.g., *ACME* [31], *oligo*, and *tilingArray* [24]. For analyses that go beyond pairwise comparisons of samples and use more complex (multi-)factorial experimental designs or retrospective studies of collections of tissues from patients, the package *limma* [20] offers a powerful statistical modeling interface and facilitates computation of appropriate reporter-wise statistics.

We also demonstrated a few conceivable follow-up investigations. Bioconductor allows for easy integration of ChIP-chip results with other resources, such as annotated genome elements, gene expression data, or DNA–protein interaction networks.

### Software Versions

This tutorial was generated using the following package versions:

- R version 2.8.0 Under development (unstable) (2008-09-13 r46541), x86_64-unknown-linux-gnu
- Locale: LC_CTYPE = en_US.ISO-8859-1;LC_NUMERIC = C;LC_TIME = en_US.ISO-8859-1;LC_COLLATE = en_US.ISO-8859-1;LC_MONETARY = C;LC_MESSAGES = en_US.ISO-8859-1;LC_PAPER = en_US.ISO-8859-1;LC_NAME = C;LC_ADDRES8859-1;LC_IDENTIFICATION = C
- Base packages: base, datasets, graphics, grDevices, methods, splines, stats, tools, utils
- Other packages: affy 1.19.4, affyio 1.9.1, annotate 1.19.2, AnnotationDbi 1.3.9, Biobase 2.1.7, biomaRt 1.15.1, ccTutorial 0.9.5, codetools 0.2-1, DBI 0.2-4, digest 0.3.1, fortunes 1.3-5, genefilter 1.21.3, geneplotter 1.19.5, GO.db 2.2.3, graph 1.19.5, lattice 0.17-15, limma 2.15.11, preprocessCore 1.3.4, RColorBrewer 1.0-2, RCurl 0.9-4, Ringo 1.5.13, RSQLite 0.7-0, SparseM 0.78, survival 2.34-1, topGO 1.9.0, vsn 3.7.6, weaver 1.7.0, xtable 1.5-3
- Loaded via a namespace (and not attached): cluster 1.11.11, grid 2.8.0, KernSmooth 2.22-22, XML 1.96-0

### Supporting Information

Text S1 Analyzing ChIP-chip data using Bioconductor. This document contains supplementary text, source code, and figures.
(5.11 MB PDF)
