# Supplementary material for: Analyzing ChIP-chip Data Using Bioconductor
Source: PLoS Comput Biol. 2008 Nov 28;4(11):e1000227. doi: 10.1371/journal.pcbi.1000227 (PMC2582686; doi:10.1371/journal.pcbi.1000227)
Supplement: Text S1 — Analyzing ChIP-chip data using Bioconductor. This document contains supplementary text, source code, and figures. (5.11 MB PDF) [file pcbi.1000227.s001.pdf]

Supplement: Analyzing ChIP-chip data using Bioconductor

Joern Toedling, Wolfgang Huber

Contents

1 Introduction **2**

2 Importing the data into R **2**

3 Quality assessment **4**

4 Mapping reporters to the genome **8**

4.1 Average spacing between reporter mach positions 10

5 Genome annotation **10**

6 Preprocessing **13**

7 Preprocessed reporter intensities around the gene *Crmp1* **14**

8 Smoothing of reporter intensities **14**

9 Alternative methods for finding ChIP-enriched regions **16**

10 Comparing ChIP-enrichment between the tissues **18**

10.1 Enriched-region-wise comparison 18

11 ChIP results and expression microarray data **21**

11.1 Preprocess the microarray expression data 21

11.2 Map Ensembl identifier to Affymetrix probe sets 21

1 Introduction

This document supplements the manuscript "Analyzing ChIP-chip data using Bioconductor". The manuscript demonstrates how to use the tools R and Bioconductor for a ChIP-chip data analysis.

The R software can be obtained by following the installation instructions at `http://www.r-project.org`.

For obtaining the Bioconductor packages that are needed for redoing this analysis, we recommend `biocLite` function from the Bioconductor web site. Follow these steps from within R to install the required package (the data package *ccTutorial* is > 300 MB in size).

```
> source("http://www.bioconductor.org/biocLite.R")
> biocLite(c("Ringo", "biomaRt", "topGO", "ccTutorial"))
```

Further information about the installation of Bioconductor packages can be found at `http://www.bioconductor.org/docs/install`.

```
> library("Ringo")
> library("biomaRt")
> library("topGO")
> library("ccTutorial")
```

This document has been written in the `Sweave` [1] format, which combines explanatory text and the R source code that has been used in this analysis. One advantage of this format is that the analysis can easily be reproduced by the reader. The R package *ccTutorial* contains all the data and scripts used in this manuscript.

2 Importing the data into R

The provided data are measurements of enrichment for H3K4me3 in heart and brain cells. For each microarray, the scanning output consists of two files, one holding the Cy3 intensities (the untreated *input* sample), the other one the Cy5 intensities from the immunoprecipitated sample. These files are tab-delimited text files in NimbleGen's *pair* format.

The microarray platform was a set of 4 arrays containing about 390k reporters each and meant to tile selected promoter regions in the *Mus musculus* genome (assembly *mm5*) with one base every 100bp. Thus for every sample, we have 8 files (4 arrays $\times$ 2 dyes).

```
> pairDir <- system.file("PairData",package="ccTutorial")
> list.files(pairDir, pattern="pair$")
```

```
 [1] "47101_532.pair" "47101_635.pair" "48153_532.pair" "48153_635.pair"
 [5] "48158_532.pair" "48158_635.pair" "48170_532.pair" "48170_635.pair"
 [9] "48175_532.pair" "48175_635.pair" "48180_532.pair" "48180_635.pair"
[13] "48182_532.pair" "48182_635.pair" "49728_532.pair" "49728_635.pair"
```

In addition, there is one text file for each array type that holds details on the samples, including which two *pair* files belong to which sample.

```
> read.delim(file.path(pairDir,"files_array1.txt"), header=TRUE)
```

```
  SlideNumber    FileNameCy3    FileNameCy5                      DESIGN_NAME
1       48153 48153_532.pair 48153_635.pair 2005-06-17_Ren_MM5Tiling_Set1
2       48170 48170_532.pair 48170_635.pair 2005-06-17_Ren_MM5Tiling_Set1
  SAMPLE_SPECIES   Cy3     Cy5 Tissue
1   Mus_musculus input H3K4me3  brain
2   Mus_musculus input H3K4me3  heart
```

The columns `FileNameCy3` and `FileNameCy5` hold which of the raw data files belong to which sample. The immunoprecipitated extract was tagged with the Cy5 dye in the experiment; so the column `Cy5` holds which antibody has been used for the immunoprecipitation, in this case one against the histone modification `H3K4me3`.

The file `spottypes.text` describes the reporter categories on the array (such Spot Types files are also used in the Bioconductor package *limma* [2]).

From these files, we can read in the raw reporter intensities and obtain four objects of class *RGList*, a class defined in package *limma*. Each object contains the readouts from all samples measured on the same array type.

```
> RGs <- lapply(sprintf("files_array%d.txt",1:4),
+   readNimblegen, "spottypes.txt", path=pairDir)
```

An *RGList* object is a list and contains the raw intensities of the two hybridizations for the red and green channel plus information about the reporters on the array and the analyzed samples.

```
> head(RGs[[1]]$R)
```

```
     48153_635 48170_635
[1,]  18680.45  27445.45
[2,]  19510.55  28003.45
[3,]  19269.45  26622.55
[4,]    348.67  24435.33
[5,]    312.78    214.00
[6,]    348.89  25187.22
```

```
> head(RGs[[1]]$G)
```

```
     48153_532 48170_532
[1,]  65535.00  65535.00
[2,]  65535.00  65535.00
[3,]  62598.89  65535.00
[4,]   1197.89  65535.00
[5,]   1433.00   1781.56
[6,]   1608.56  61490.45
```

```
> tail(RGs[[1]]$genes)

       GENE_EXPR_OPTION        PROBE_ID POSITION   X   Y Status             ID
390606              MM5 MM5000P03209684 52759608 140  32  Probe MM5000P03209684
390607              MM5 MM5000P03370281 82709533 146 534  Probe MM5000P03370281
390608              MM5 MM5000P03410577 89884304  33 495  Probe MM5000P03410577
390609              MM5 MM5000P03428135 93306337 404 786  Probe MM5000P03428135
390610              MM5 MM5000P03152702 42800575 548 836  Probe MM5000P03152702
390611              MM5 MM5000P03113901 35200666 687 907  Probe MM5000P03113901
```

Among the read-in values are those coming from reporters[1] matching the genome sequence as well as some from the manufacturer's "control" reporters on the array.

```
> table(RGs[[1]]$genes$Status)

  H_Code Negative    Probe   Random   V_Code
      40     2063   373896    14572       40
```

The *RGList* is a common class for raw two-color data. Thus, the following steps can easily be applied to other, non-NimbleGen microarrays, which for example can be read in into R using *limma*'s function `read.maimages`.

3 Quality assessment

First, we look at the spatial distribution of the intensities on the array. This is useful for detecting obvious artifacts on the array, such as scratches, bright spots, finger prints etc., which may render parts or all of the readouts of one hybridization useless.

For demonstration, we first show three array surface plots with artifacts. These three microarrays were generated for another ChIP-chip study for histone modifications [3]. See the spatial distribution plots of these arrays in Figure S1. The coordinates in the picture correspond to coordinates on the surface of the microarray. The color of the dots represents the value of the raw reporter intensity, with brighter shades of green corresponding to higher intensities. For well-hybridized microarrays, a homogeneous picture can be expected. Two of the displayed three arrays show strong artifacts. The array in the left panel shows two distinct problems. The bright rim on the picture suggests that all reporters near the rim of the array high raw intensities. The second artifact is the wave pattern on the surface. This effect is known as a Moiré pattern in image processing and emerged during the scanning process of the microarray. The spatial distribution of Cy5 channel intensities on this array looks homogeneous (data not shown), which provides more evidence that the artifacts in this plot are likely due to errors in scanning the Cy3 intensities of this array. The array in the middle panel shows a large artifact that only affects the right side of the array. The

[1] the misleading slot name "genes" is due to historical reasons, dating back to the time when cDNA microarrays were mostly used to measure gene expression. In our case, each reporter is not associated to one gene but to one or more genomic locations.

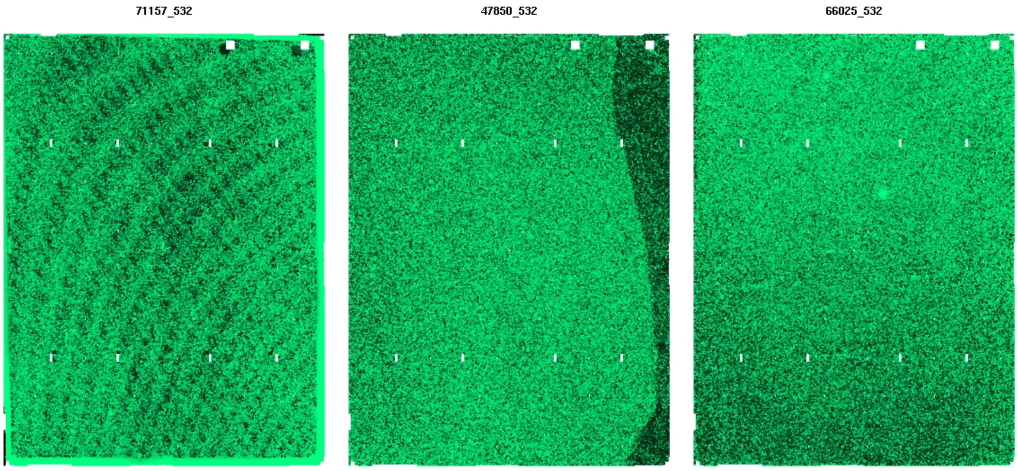

Figure S1: *Spatial distribution of reporter intensities on microarrays from another ChIP-chip study [3]. Coordinates in the picture correspond to coordinates on the surface of the microarray. The color of the dots represents the value of the raw reporter intensity, with brighter shades of green corresponding to higher intensities. For well-hybridized microarrays, a homogeneous picture can be expected.The two arrays in the left and the middle panel show strong artifacts and were excluded from later analyses. The array in the right panel shows weak artifacts and was kept for later analysis.*

array in the right panel finally shows a bright spot in the center of the array and slightly higher intensities in the upper half of the array than in the lower array. However, these artifacts are mild in comparison with the other two arrays. The array on the right side was kept for further analysis in the study, while the two on the left side and in the middle were replaced by newly hybridized arrays.

For the data of Barrera *et al.* [4], we construct one picture showing the spatial distributions for all arrays and both channels.

```
> RG1breaks <- c(0,quantile(RGs[[1]]$G, probs=seq(0,1,by=0.1)),2^16)
> jpeg("ccTutorialArrayImages.jpg", quality=100,
+      height=1074*1.5, width=768*1.5)
> par(mar=c(0.01,0.01,2.2,0.01))
> layout(matrix(c(1,2,5,6,3,4,7,8,9,10,13,14,11,12,15,16),
+        ncol=4,byrow=TRUE))
> for (this.set in 1:4){
+   thisRG <- RGs[[this.set]]
+   for (this.channel in c("green","red")){
+     my.colors <- colorRampPalette(c("black",paste(this.channel,c(4,1),
+                                    sep="")))(length(RG1breaks)-1)
+     for (arrayno in 1:2){
+       image(thisRG, arrayno, channel=this.channel,
+             mybreaks=RG1breaks, mycols=my.colors, frame.plot=FALSE)
+       mtext(side=3, line=0.2, font=2, text=colnames(thisRG[[toupper(
+             substr(this.channel,1,1))]])[arrayno])
+ }}}
> dev.off()
```

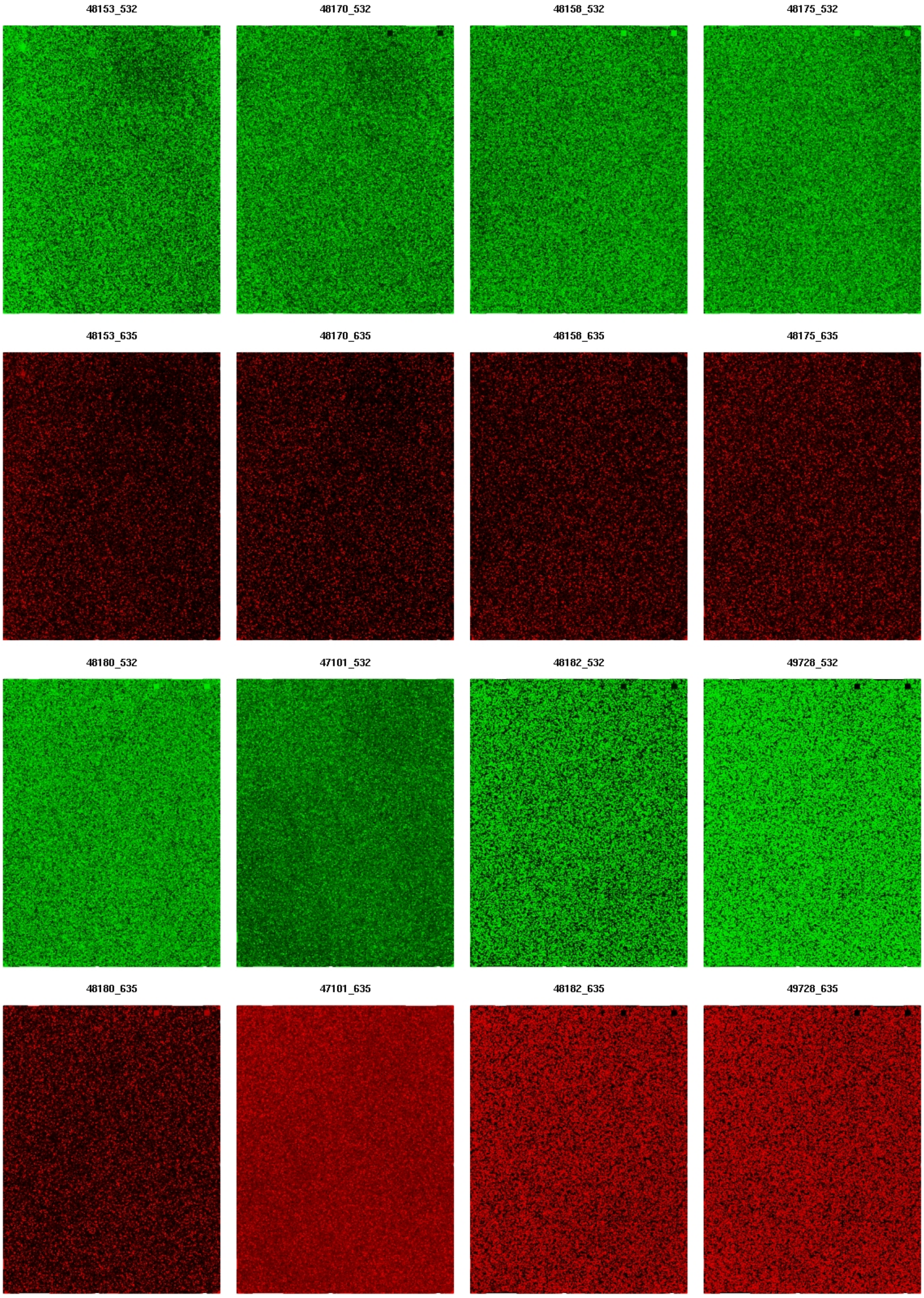

Figure S2: *Spatial distribution of raw reporter intensities laid out by the reporter position on the microarray surface. Each pair of one green and one red image on top of each other are the Cy3 and Cy5 readouts of the same hybridized microarray.*

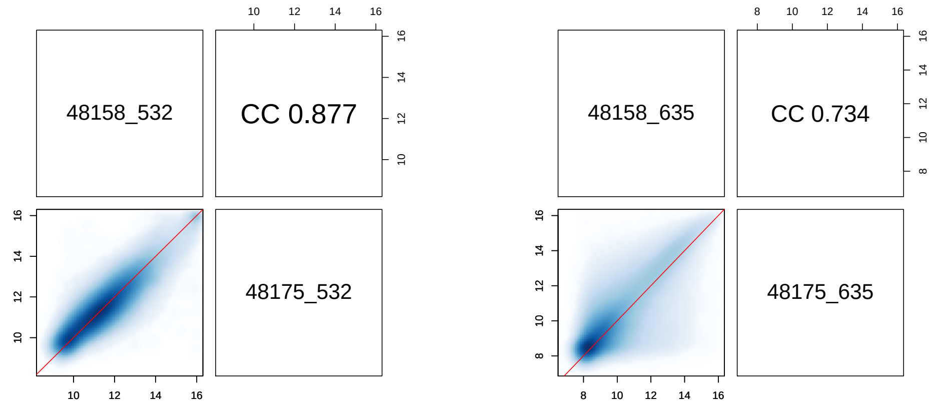

(a) (b)

Figure S3: *Scatterplot and Spearman correlation of the raw intensities from the two microarrays for* (a) *the Cy3 channel, the genomic input samples* (b) *the Cy5 channel, the H3K4me3-ChIP sample for* M. musculus *brain and heart cells.*

See Figure S2 for the images. Minor artifacts can be seen. The arrays of the first set (48153 and 48172) show a blotch of lower intensities in the upper right area of the array. These artifacts affect only a small part of the array and thus probably have a negligible effect on the results. The reporters in those affected areas of the array will yield meaningless readouts but enriched regions will be determined based on a set of multiple reporters that are distributed over the microarray surface and not on single reporters only. Also a few arrays are brighter than others, which indicate higher raw intensities for the respective arrays. These effects could be due to a larger amounts of DNA being hybridized. The scaling step during preprocessing later on is able to correct for such shifts.

On all arrays in our set, the Cy3 channel holds the intensities from the untreated *input* sample, and the Cy5 channel holds the ChIP result for heart and heart, respectively. We investigate whether this experiment setup is reflected in the reporter intensity correlation per channel (see Figure S3). Compare these two plots:

```
> corPlot(log2(RGs[[2]]$G))
```

```
> corPlot(log2(RGs[[2]]$R))
```

See Figure S3 for plots comparing the two arrays. In the scatter plots of raw reporter intensities, the fraction of dots at the diagonal is higher for the *input* samples than for the ChIP samples. Concordantly, the correlation between the intensities of the *input* samples is higher than between the ChIP samples (0.877 versus 0.734).

We also show the same plots for two of the previous arrays with artifacts (left and right panels in Figure S1). With these arrays, the Cy3 channel also holds the *input* samples, while the Cy5 channel are the ChIP samples.

These correlation plots are shown in Figure 3. The *input* (Cy3) intensities do not show any correlation, while the ChIP intensities of these samples show a much better correlation.

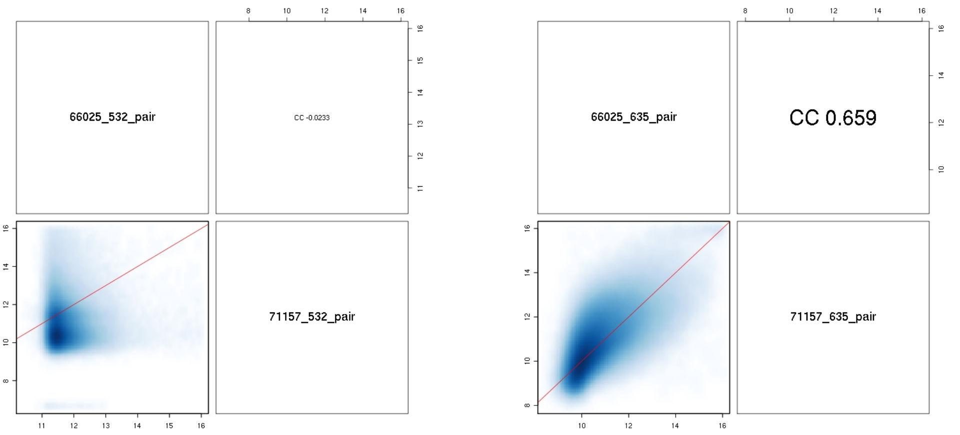

(a) (b)

Figure S4: *Scatterplot and Spearman correlation of the raw intensities from two microarrays with artifacts (see Figure S1 for* (a) *the Cy3 channel, the genomic input samples* (b) *the Cy5 channel, ChIP sample for* M. musculus *brain and heart cells.*

This is unexpected, since the ChIP samples used antibodies against different histone modifications (H4ac and H3K4me2), while the *input* samples are both genomic DNA from the same cell type (cell line C2C12).

4 Mapping reporters to the genome

A mapping of reporters to genomic coordinates is usually provided by the array manufacturer. For several reasons, however, remapping the reporter sequences to the genome may be useful. Here, the microarray had been designed on an outdated assembly of the mouse genome (mm5, May 2004). The reporter sequences were remapped to the current, almost final assembly of the mouse genome (mm9, July 2007). Remapping also allows you to specify custom criteria for what degree of sequence identity you require for a match and for uniqueness of a match.

We extracted the reporter nucleotide sequences from the downloaded NDF (NimbleGen Design Files) files. We re-mapped the reporter sequences to the genome, using the alignment tool *Exonerate* [5]. We required 97% sequence identity for a match[2]. Since the reporters on the microarray were 50mers, a sequence identity of 97% corresponds to one mismatch at most. The implicit assumption is that if 49 out of 50 nucleotides are complimentary that would be sufficient for hybridization. We did not consider a more complex hybridisation model, in which the position of the mismatch in the reporter sequence and its impact on secondary structure formation are taken into account. However, with one mismatch per 50mer, there is always a perfert match segment of ≥ 25 nucleotides in length. A matching segment of 18 or more nucleotides in length was reported to be sufficient for hybridization [6].

[2] Remapping 1.5 million reporters took about 100 processor hours on an AMD Opteron Processor 275.

Exonerate was run matching the reporter sequences in the Fasta file `RenMM5TilingProbe-Sequences.fsa` against each chromosome's sequence using the shell script `runExonerate.sh` and then condensing the per-chromosome output files into one single file using the Perl script `condenseExonerateOutput.pl`[3].

From this result file, we construct an object of class *probeAnno* to store the mapping between reporters and genome positions.

```
> probeAnno <- posToProbeAnno(file.path(system.file("exonerateData",
+   package="ccTutorial"), "allChromExonerateOut.txt"))
> allChrs <- chromosomeNames(probeAnno)
```

```
> genome(probeAnno) <- "M. musculus (mm9)"
> arrayName(probeAnno) <- "2005-06-17_Ren_MM5Tiling"
```

```
> show(probeAnno)
```

```
A 'probeAnno' object holding the mapping between
reporters and genomic positions.
Chromosomes: 10 11 12 13 14 15 16 17 18 19 1 2 3 4 5 6 7 8 9 X Y
Microarray platform: 2005-06-17_Ren_MM5Tiling
Genome: M. musculus (mm9)
```

```
> ls(probeAnno)
```

```
 [1] "10.end"    "10.index"  "10.start"  "10.unique" "11.end"    "11.index"
 [7] "11.start"  "11.unique" "12.end"    "12.index"  "12.start"  "12.unique"
[13] "13.end"    "13.index"  "13.start"  "13.unique" "14.end"    "14.index"
[19] "14.start"  "14.unique" "15.end"    "15.index"  "15.start"  "15.unique"
[25] "16.end"    "16.index"  "16.start"  "16.unique" "17.end"    "17.index"
[31] "17.start"  "17.unique" "18.end"    "18.index"  "18.start"  "18.unique"
[37] "19.end"    "19.index"  "19.start"  "19.unique" "1.end"     "1.index"
[43] "1.start"   "1.unique"  "2.end"     "2.index"   "2.start"   "2.unique"
[49] "3.end"     "3.index"   "3.start"   "3.unique"  "4.end"     "4.index"
[55] "4.start"   "4.unique"  "5.end"     "5.index"   "5.start"   "5.unique"
[61] "6.end"     "6.index"   "6.start"   "6.unique"  "7.end"     "7.index"
[67] "7.start"   "7.unique"  "8.end"     "8.index"   "8.start"   "8.unique"
[73] "9.end"     "9.index"   "9.start"   "9.unique"  "X.end"     "X.index"
[79] "X.start"   "X.unique"  "Y.end"     "Y.index"   "Y.start"   "Y.unique"
```

For each genomic position matched by a reporter, it was recorded whether it was the sole match position of that reporter in the genome or whether that reporter also matched other genomic positions[4]. For example, how many reporter-match positions on chromosome 9 are

[3]all the scripts mentioned here are included in the `scripts` directory of the package

[4]More precisely, a reporter matches a 25-nucleotide "segment". That segment, however, is uniquely defined by the genomic position of its central nucleotide, which is why I refer to the "position" that is matched by a reporter.

unique matches of those reporters (code: 0) and how many are matched by reporters that have multiple matches in the genome (code:3)?

```
> table(probeAnno["9.unique"])

    0     3
65556 11018
```

The majority of match positions are unique reporter match positions. The intensities of reporters matching multiple genomic locations will be excluded from later analysis (smoothing, identification of ChIP-enriched regions), since the readouts of these reporters are ambiguous.

4.1 Average spacing between reporter mach positions

```
> startDiffByChr <- lapply(as.list(allChrs), function(chr){
+   chrsta <- probeAnno[paste(chr,"start",sep=".")]
+   chruni <- probeAnno[paste(chr,"unique",sep=".")]
+   ## get start positions of unique reporter match positions
+   return(diff(sort(chrsta[chruni=="0"])))})
> startDiff <- unlist(startDiffByChr, use.names=FALSE)
> table(cut(startDiff, breaks=c(0,50,99,100,200,1000,max(startDiff))))

          (0,50]           (50,99]          (99,100]         (100,200]
            1995              2323           1150525             78846
     (200,1e+03] (1e+03,7.65e+06]
          132674             34888
```

The majority of unique reporter match positions have an offset of 100 bp between their start positions.

5 Genome annotation

Later on, found enriched regions will be related to annotated genome features, such as gene start and end positions. Using the Bioconductor package *biomaRt* [7], we can obtain an up-to-date annotation of the mouse genome from the Ensembl data base [8].

```
> ensembl  <- useMart("ensembl", dataset="mmusculus_gene_ensembl")
> gene.ids <- unique(unlist(lapply(as.list(c(1:19,"X","Y")),
+    function(this.chr)
+     getBM(attributes="ensembl_gene_id", filters="chromosome_name",
+           values=this.chr, mart=ensembl)[,1]), use.names=FALSE))
>     sel.attributes=c("ensembl_gene_id", "mgi_symbol", "chromosome_name",
+           "strand", "start_position","end_position", "description")
> mm9genes <- getBM(attributes=sel.attributes, filters="ensembl_gene_id",
+                  value=gene.ids, mart=ensembl)
```

For later use, we replace the formal element names retrieved from the data base by simpler ones.

```
> mm9genes$name    <- mm9genes$"ensembl_gene_id"
> mm9genes$gene    <- mm9genes$"ensembl_gene_id"
> mm9genes$chr     <- mm9genes$chromosome_name
> mm9genes$symbol  <- mm9genes$"mgi_symbol"
> mm9genes$start   <- mm9genes$"start_position"
> mm9genes$end     <- mm9genes$"end_position"
> mm9genes$feature <- rep("gene",nrow(mm9genes))
```

Some genes occur in multiples in the table because an Ensembl gene can have more than one MGI Symbol defined for it. We keep allow only one row in the table per gene and append additional MGI symbols to the *description* element of each gene.

```
> if (any(duplicated(mm9genes$name))){
+   dupl <- unique(mm9genes$name[duplicated(mm9genes$name)])
+   G <- lapply(as.list(dupl), function(this.gene){
+     this.gff <- subset(mm9genes,name == this.gene)
+     if (nrow(unique(this.gff[,c("name","chr","start","end",
+         "description")]))>1) return(this.gff[1,,drop=FALSE])
+     non.zero.gff <- subset(this.gff, nchar(symbol)>0)
+     this.other.sym <- NULL
+     if (nrow(non.zero.gff)> 0){
+       shortest <- which.min(nchar(non.zerogff$symbol))
+       this.new.sym <- non.zero.gff$symbol[shortest]
+       if (nrow(non.zero.gff)>1)
+         this.other.sym <- paste("Synonyms",
+            paste(non.zero.gff$symbol[-shortest],collapse=","),sep=":")
+     } else { this.new.sym <- "" }
+     this.gff$symbol[1] <- this.new.sym
+     if (!is.null(this.other.sym))
+       this.gff$description[1] <- paste(this.gff$description[1],
+                                  this.other.sym,sep=";")
+     return(this.gff[1,,drop=FALSE])
+   })
+   mm9genes <- rbind(mm9genes[-which(mm9genes$name %in% dupl),],
+                     do.call("rbind",G))
+ }
```

Finally, we reorder the table rows by gene chromosome and start position.

```
> mm9genes <- mm9genes[order(mm9genes$chr, mm9genes$start),
+   c("name","chr","strand","start","end","symbol","description","feature")]
> rownames(mm9genes) <- NULL
```

The resulting table holds the coordinates, Ensembl gene identifiers, MGI symbols, and description of all the genes annotated for the *mm9* mouse assembly. Have a look at a few example lines from the table.

```
> mm9genes[sample(seq(nrow(mm9genes)),4),
+   c("name", "chr", "strand", "start", "end", "symbol")]
```

```
                    name chr strand     start       end        symbol
7284  ENSMUSG00000057903  14      1  51044196  51045125       Olfr739
10209 ENSMUSG00000039615  17     -1  25967581  25970306         Stub1
15715 ENSMUSG00000068823   3      1 102824530 102862108         Csde1
24914 ENSMUSG00000006241   9      1  21731915  21740316 2510048L02Rik
```

We also retrieve the Gene Ontology (GO, [9]) annotation for each gene, but discard those annotations that have only been *inferred from electronic annotation* (evidence code: IEA), are based on a *non-traceable author statement* (NAS) or for which there is *no biological data* (ND) available.

```
> ensembl  <- useMart("ensembl", dataset="mmusculus_gene_ensembl")
> ontoGOs <- lapply(as.list(c("biological_process","cellular_component",
+                    "molecular_function")), function(onto){
+   ontoBM <- getBM(mart=ensembl, attributes=c("ensembl_gene_id",
+                    paste("go",onto,"id", sep="_"),
+                    paste("go",onto,"linkage_type", sep="_")),
+                   filters="ensembl_gene_id", value=mm9genes$name)
+   names(ontoBM) <- c("ensembl_gene_id","go","evidence_code")
+   ontoBM <- subset(ontoBM,!( evidence_code %in% c("","IEA","NAS","ND")))
+ })
> mm9GO <- do.call("rbind", ontoGOs)
```

```
> mm9.gene2GO <- with(mm9GO, split(go, ensembl_gene_id))
```

Finally, we create a mapping of gene identifiers to reporters that had been mapped into the gene or its upstream region.

```
> mm9.g2p <- features2Probes(gff=mm9genes, probeAnno=probeAnno)
```

```
> table(cut(listLen(mm9.g2p),breaks=c(-1,0,10,50,100,500,1200)))
```

```
       (-1,0]        (0,10]       (10,50]      (50,100]     (100,500]
        10790           640          7341          5949          2675
(500,1.2e+03]
           39
```

This last table shows how many genes have that number of reporters mapped into their upstream region or inside of them. The numbers of reporters are given in open interval notation with, e.g., (10,50] meaning 11 to 50 reporters.

For later use, we determine which genes have a sufficient number - arbitrarily we say 5 - of reporters mapped to their upstream region or inside of them. We also determine which of these genes have been annotated with at least one GO term.

```
> arrayGenes <- names(mm9.g2p)[listLen(mm9.g2p)>=5]
> arrayGenesWithGO <- intersect(arrayGenes, names(mm9.gene2GO))
```

6 Preprocessing

We derive $\log_2$ fold changes Cy5/Cy3 for each reporter and scale these by subtracting Tukey's biweight mean from each log2 ratio, the standard scaling procedure suggested by NimbleGen. We only perform this scaling procedure, since we are not aware of any normalization method that is completely appropriate for ChIP-chip with antibodies against histone modifications. One common assumption of many normalization methods is that the variation of almost all reporter levels does not reflect biological variation between samples/conditions (*input*, ChIP) but is non-biological variation, *e. g.*, due to differences in sample processing and hybridization. This assumption probably does not hold in this case, as the fraction of histones bearing post-translational modifications cannot safely be assumed to be small.

Each of the four microarrays used contains a unique set of reporters. Thus, we preprocess the arrays separately by type and only then combine the results into one object holding the preprocessed readouts for all reporters.

```
> MAs <- lapply(RGs, function(thisRG)
+   preprocess(thisRG[thisRG$genes$Status=="Probe",],
+              method="nimblegen", returnMAList=TRUE))
> MA <- do.call("rbind",MAs)
> X  <- asExprSet(MA)
> sampleNames(X) <- paste(X$Cy5, X$Tissue, sep=".")
```

The result is an object of class *ExpressionSet*, the Bioconductor class for storing preprocessed microarray data. Note that first creating an *MAList* for each array type, combining them with `rbind` and then converting the result into an *ExpressionSet* is only necessary if the reporters are distributed over more than one microarray design (four in this case).

```
> show(X)
```

```
ExpressionSet (storageMode: lockedEnvironment)
assayData: 1495582 features, 2 samples
  element names: exprs
phenoData
  sampleNames: H3K4me3.brain, H3K4me3.heart
  varLabels and varMetadata description:
    SlideNumber: NA
    FileNameCy3: NA
    ...: ...
    Tissue: NA
    (8 total)
  additional varMetadata: varLabel
featureData
  featureNames: 16716, 16717, ..., 3906113  (1495582 total)
  fvarLabels and fvarMetadata description:
    GENE_EXPR_OPTION: NA
    PROBE_ID: NA
    ...: ...
```

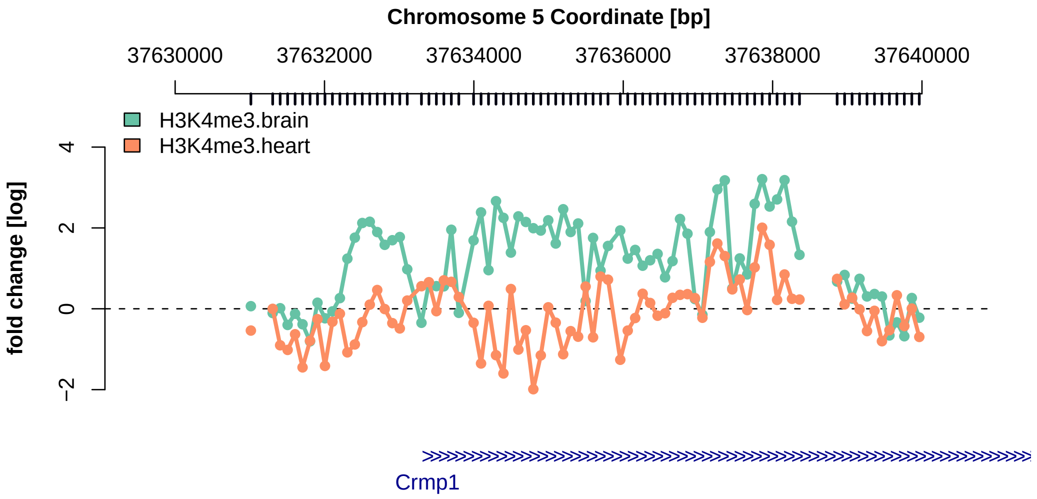

Figure S5: *Normalized reporter intensities for H3K4me3 ChIP around the TSS of the Crmp1 gene in* M. musculus *brain and heart cells. The ticks below the genomic coordinate axis on top indicate genomic positions matched by reporters on the microarray. The blue arrows on the bottom mark the Crmp1 gene with the arrow direction indicating that the gene is located on the Watson strand.*

```
    ID: NA
    (7 total)
experimentData: use 'experimentData(object)'
Annotation:
```

7 Preprocessed reporter intensities around the gene *Crmp1*

We visualize the preprocessed H3K4me3 ChIP-chip reporter-wise readouts around the start of the *Crmp1* gene. H3K4me3 has frequently been shown to be associated to active transcription (e. g. , [3]) and the gene *Crmp1* has been reported as being expressed in brain cells [10].

```
> chipAlongChrom(X, chrom="5", xlim=c(37.63e6,37.64e6), probeAnno=probeAnno,
+                gff=mm9genes, ylim=c(-3,5), paletteName="Set2")
```

See the result in Figure S5. In brain cells, the intensities for enrichment of H3K4me3 around the gene's start position tend to be positive, while the signal for heart cells is around or below zero.

8 Smoothing of reporter intensities

To ameliorate specific reporter effects as well as the stochastic noise, we perform a smoothing over individual reporter intensities before looking for ChIP-enriched regions. A window of 900 bp width is slided along the chromosome, and the reporter level at genomic position x_0

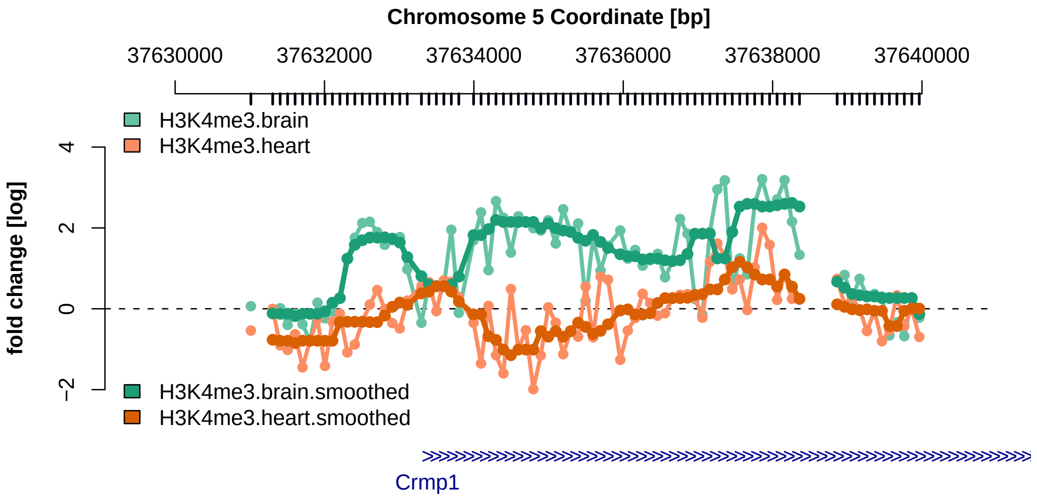

Figure S6: *Normalized and smoothed reporter intensities for H3K4me3 ChIP around the TSS of the gene* Crmp1 *in* M. musculus *brain and heart cells.*

is replaced by the median over the intensities of those reporters mapped inside the window centered at x_0.

Factors taken into account in the choice of the sliding-window width were the size distribution of DNA fragments after sonication (commonly around 1 kbp) and the spacing between reporter matches on the genome (mostly 100 bp). We chose a window-width of 900 bp, which was slightly less than the average fragment size and meant that most windows included >= 5 reporters. With this window width, we could be sure that the signal is not smoothed over many fragments and was calculated as the median over ≥ 5 reporters. At any position x_0 at which the window comprised less than three reporter-matched positions, the smoothed level was flagged as missing, as the data was insufficient to provide information about ChIP enrichment at such a position.

```
> smoothX <- computeRunningMedians(X, probeAnno=probeAnno,
+    modColumn="Tissue", allChr=allChrs, winHalfSize=450, min.probes=5)
> sampleNames(smoothX) <- paste(sampleNames(X),"smoothed",sep=".")
```

Compare the smoothed reporter intensities with the non-smoothed ones around the start of the gene *Crmp1*.

```
> chipAlongChrom(X, chrom="5", xlim=c(37.63e6,37.64e6), probeAnno=probeAnno,
+                 gff=mm9genes, paletteName="Set2", ylim=c(-3,5))
> chipAlongChrom(smoothX, chrom="5", xlim=c(37.63e6,37.64e6), add=TRUE,
+                 probeAnno=probeAnno,ilwd=4, paletteName="Dark2")
```

See Figure S6 for a comparison of the original and smoothed reporter levels around the gene *Crmp1*.

9 Alternative methods for finding ChIP-enriched regions

We have presented the algorithm of package *Ringo* for finding Chip-enriched regions in ChIP-Chip against H3K4me3. There are important differences between ChIP-chip against histone modifications and ChIP-chip against transcription factors. With transcription factors, it is safe to assume that the majority of genomic regions will not show a real binding site for that transcription factor. Hence, most reporters on the microarray will not indicate true enrichment, at least not when the tiling microarrays represent the whole genome or an unbiased subset of the genome. This situation is beneficial for the data preprocessing and for identifying ChIP-enriched regions, since most of the data can safely be assumed to show non-enrichment. With histone modifications, on the other hand, the degree and extent to which the genome shows a certain histone modification can only be guessed at the present time. This situation make estimation of the background distribution of reporter levels under non-enrichment difficult.

Moreover, transcription factor binding sites are highly localized point effects, meaning that the transcription factor binds at one specific position directly or indirectly to the DNA and the signal will show a peak shape around this position. The highest point of the signal peak will be as close to the actual binding site as the reporter-tiling on the microarray allows (see [11] for an extended discussion and for a derived model of TF ChIP-chip data). With histone modifications, the enzyme which modifies the histone tail is unlikely to act on only one single histone protein, but will modify a number of nearby histones. A single-nucleosome resolution study of histone modifications in *Saccharomyces cerevisiae* has shown that modifications occur in the form of broad modified domains and that adjacent nucleosomes mostly share the same modifications [12].

Many other suggested algorithms for finding ChIP-enriched regions are based on the assumption that the fraction of reporters that show enrichment is very small and are therefore not applicable to ChIP-chip against histone modifications. However, the algorithm in *Ringo* is by no means the only suitable algorithm for this task. Users can choose to apply other algorithms that are contained in other R/Bioconductor packages. In the following, we demonstrate an example application of one other algorithm to the data.

CMARRT The package *CMARRT* [13] can be obtained from `http://www.stat.wisc.edu/~kuanp/CMARRT` . Based on the example source code in the package vignette, we use *CMARRT* to identify which regions are enriched by ChIP against H3K4me3 in brain cells.

```
> library("CMARRT")
> cmarrtDat <- do.call("rbind", lapply(as.list(allChrs), function(chr){
+   areUni <- probeAnno[paste(chr,"unique",sep=".")]==0
+   chrDat <- data.frame("chr"=rep(chr, sum(areUni)),
+       "start"=probeAnno[paste(chr,"start",sep=".")][areUni],
+       "stop"=probeAnno[paste(chr,"end",sep=".")][areUni],
+       "logR"=exprs(X)[probeAnno[paste(chr,"index",sep=".")][areUni],1])
+ }))
> cmarrtRes <- cmarrt.ma(cmarrtDat, M=0.5, frag.length=900,
+                         window.opt = "fixed.gen.dist")
> cmarrtReg <- cmarrt.peak(cmarrtRes, alpha=0.05, method="BY", minrun=4)
> cmarrtRegDf <- lapply(cmarrtReg, as.data.frame)$cmarrt.bound
> names(cmarrtRegDf)[1:3] <- c("chr","start","end")
```

These are a few of the ChIP-enriched regions for H3K4me3 in brain cells, as identified by *CMARRT*:

```
> head(cmarrtRegDf)
```

```
  chr   start     end n.probe       min.pv       ave.pv
1  10 3133409 3137495      39 2.785864e-16 6.000587e-06
2  10 4521803 4523152      14 9.585187e-09 2.532042e-05
3  10 4540005 4541530      15 7.250199e-08 6.426842e-05
4  10 4795875 4797192      13 1.393900e-11 2.201104e-05
5  10 4978403 4979752      14 2.373338e-08 3.682241e-05
6  10 5151588 5152337       8 2.276438e-09 2.914762e-05
```

We assess the overlap between ChIP-enriched regions identified by the two methods, exemplarily for chromosome 9.

```
> ringoChersChr9 <- subset(chersXD, chr=="9" & cellType=="brain")
> cmarrtChersChr9 <- subset(cmarrtRegDf, chr=="9")
> dim(ringoChersChr9)
```

```
[1] 675   9
```

```
> dim(cmarrtChersChr9)
```

```
[1] 639   6
```

The overlap between two ChIP-enriched regions $R_{i,Ringo}$ and $R_{j,CMARRT}$, which were identified by *Ringo* and *CMARRT*, respectively, is computed as

$$\text{Ov}\left(R_{i,Ringo}, R_{j,CMARRT}\right) = \frac{\text{length}\left(R_{i,Ringo} \cap R_{j,CMARRT}\right)}{\min\left(\text{length}(R_{i,h_1}), \text{length}(R_{j,h_2})\right)} \tag{1}$$

where "$\cap$" denotes region intersection and length(R_i) is the length of region R_i in nucleotides.

```
> chersChr9Overlap <- as.matrix(
+    regionOverlap(ringoChersChr9, cmarrtChersChr9))
> minRegChr9Len <- outer(with(ringoChersChr9, end-start+1),
+                        with(cmarrtChersChr9, end-start+1), pmin)
> fracChr9Overlap <- chersChr9Overlap /minRegChr9Len
```

```
> summary(apply(fracChr9Overlap, 1, max))
```

```
   Min. 1st Qu.  Median    Mean 3rd Qu.    Max. 
 0.0000  0.9737  0.9900  0.9086  1.0000  1.0000 
```

One average, a ChIP-enriched region identified by *Ringo* is overlapped to $\approx$ 91% by a ChIP-enriched region identified by *CMARRT*. The identified regions are highly consistent between the two methods.

10 Comparing ChIP-enrichment between the tissues

First, we have taken a gene-centric position and consider which genes are associated to each tissue specifically.

```
> brainGenes <- getFeats(chersX[sapply(chersX, cellType)=="brain"])
> heartGenes <- getFeats(chersX[sapply(chersX, cellType)=="heart"])
> brainOnlyGenes <- setdiff(brainGenes, heartGenes)
> heartOnlyGenes <- setdiff(heartGenes, brainGenes)
```

10.1 Enriched-region-wise comparison

We compute the base-pair overlap between enriched regions found in brain cells with those found in heart cells. We define that region R_i is defined to *overlap* with region R_j if

$$\text{length}(R_i \cap R_j) \quad \geq \quad 0.7 \cdot \min(\text{length}(R_i), \text{length}(R_j)) \tag{2}$$

where $\text{length}(R_i)$ denotes the length of region R_i in nucleotides. We define an enriched region as tissue-specific if it does not overlap with any region from another tissue according to the definition above.

```
> brainRegions <- subset(chersXD, cellType=="brain")
> heartRegions <- subset(chersXD, cellType=="heart")
> chersOBL <- as.matrix(regionOverlap(brainRegions, heartRegions))
> minRegLen <- outer(with(brainRegions, end-start+1),
+                    with(heartRegions, end-start+1), pmin)
> fracOverlap <- chersOBL/minRegLen
```

```
> brainSpecReg <-  brainRegions[rowMax(fracOverlap)<0.7,]
> heartSpecReg <-  heartRegions[rowMax(t(fracOverlap))<0.7,]
> mean(is.element(unlist(strsplit(brainSpecReg$features,
+    split="[[:space:]]"), use.names=FALSE), brainOnlyGenes))
```

```
[1] 0.799639
```

```
> selGenes <- intersect(unlist(strsplit(brainSpecReg$features,
+    split="[[:space:]]"), use.names=FALSE), heartGenes)
```

Note that only 80% of the genes related to non-overlapping ChIP-enriched regions show such regions in brain cells only. The other genes show such regions in both tissues but their positions differ between the tissues.

We can assess whether these 690 genes show a typical positioning of H3K4me3 to each other, such as 'in heart cells they display H3K4me3 enriched regions upstream of the genes, while in brain cells the show H3K4me3 between gene start and stop coordinates'. We use the genes-to-reporters mapping that we have created earlier for this investigation.

```
> targetPos <- seq(-5000, 10000, by=250)
```

For each gene, we assess the genomic region from 5kb upstream to 10 kb downstream of the gene start.

```
> selGenesVal <- vector("list", length(selGenes))
> for (i in 1:length(selGenes)){
+   if (i %% 1000 == 0) cat(i,"... ")
+   ix <- mm9.g2p[[selGenes[i]]]
+   if (length(ix)==0) next
+   iy <- exprs(smoothX)[names(ix),,drop=FALSE]
+   if (sum(!is.na(iy[,1]))<2) next
+   iy <- apply(iy, 2, function(a)
+               approx(x=ix, y=a, xout=targetPos)$y)
+   selGenesVal[[i]] <- iy
+ }
> names(selGenesVal) <- selGenes
```

```
> quants <- c(0.1,0.3,0.5,0.7, 0.9)
> valsBySample <- lapply(list("H3K4me3.brain.smoothed",
+    "H3K4me3.heart.smoothed"), function(thisSample){
+       do.call("cbind", lapply(selGenesVal, function(theseValues){
+       return(theseValues[,thisSample,drop=FALSE])}))})
> names(valsBySample) <- c("H3K4me3.brain.smoothed",
+                          "H3K4me3.heart.smoothed")
> quantsBySample <- lapply(valsBySample, function(theseValues)
+   apply(theseValues, 1, quantile, probs=quants, na.rm=TRUE))
```

We normalize these densities of the observed fold changes by genomic positions by the densities of mapped reporters.

```
> probePosDens <- density(unlist(mm9.g2p, use.names=FALSE))
> probePosY <- approx(x=probePosDens$x, y=probePosDens$y,
+                     xout=targetPos)$y
> probePosY <- probePosY/max(probePosY)
```

```
> plot(x=0,y=0, ylim=c(-0.2,3.5), xlim=range(targetPos),
+      xaxt="n", type="n", xlab="Distance to gene start [bp]",
+      ylab="smoothed fold-change")
> axis(side=1, at=seq(-5000, 10000, by=1000),
+      labels=paste(-5:10,"kb",sep=""))
> mycolors <- c("green","orange")
> for (i in 1:2){
+   lines(x=targetPos,y=quantsBySample[[i]][3,]*probePosY,
+         lwd=2, col=mycolors[i])
+   lines(x=targetPos,y=quantsBySample[[i]][5,]*probePosY,
+         lwd=2, lty=2, col=mycolors[i])
+ }
```

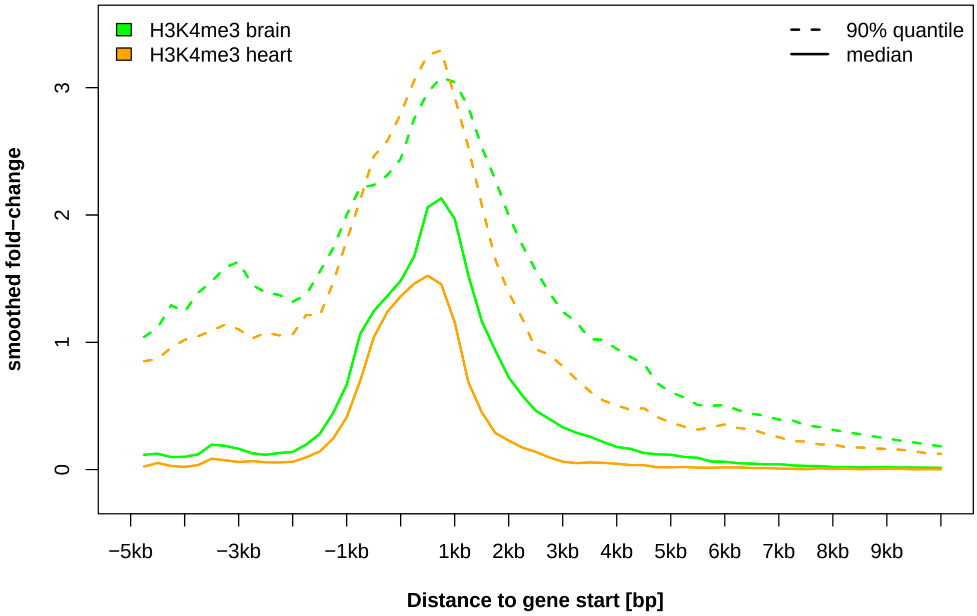

Figure S7: *Densities of selected quantiles of the smoothed fold-changes for H3K4me3 ChIP in* M. musculus *brain and heart cells for genes that show H3K4me3 enriched regions in both tissues but in separate positions.*

```
> legend(x="topleft", fill=c("green","orange"), bty="n",
+        legend=c("H3K4me3 brain", "H3K4me3 heart"))
> legend(x="topright", lty=c(2,1), lwd=2, bty="n",
+        legend=c("90% quantile", "median"))
```

See Figure S7 for the densities. There are no clear tissue-wise trends where these enriched regions are in relation to the gene start coordinate. In both tissues, the smoothed intensities on average are highest within 1kb after the gene start coordinate, while in brain cells the density shows a second, smaller cher within 1kb upstream of the gene start.

We also investigate genes that have separate enriched regions in both tissues for over-represented GO annotations.

```
> sepRegRes <- sigGOTable(selGenes=selGenes)
> print(sepRegRes)
```

GO.ID	Term	Annotated	Significant	Expected	p.value
GO:0045648	positive regulation of erythrocyte differentiation	5	4	0.26	3.5e-05
GO:0045176	apical protein localization	8	4	0.42	0.00044
GO:0007406	negative regulation of neuroblast proliferation	4	3	0.21	0.00055

Table S1: *GO terms that are significantly over-represented among genes that show different H3K4me3 regions in heart and brain cells*

See the results in Table S1.

11 ChIP results and expression microarray data

Barrera *et al.* [4] also provide expression microarray data for five their analyzed *M. musculus* tissues.

11.1 Preprocess the microarray expression data

The data were obtained from the supplementary web page to the publication [4], imported into R and preprocessed as follows.

```
> library("affy")
> library("mouse4302cdf")
> AB <- ReadAffy(celfile.path=system.file("expression",
+                                         package="ccTutorial"))
> barreraExpressionX <- mas5(AB)
> barreraExpressionX$Tissue <- sapply(
+    strsplit(sampleNames(barreraExpressionX),split="\\."),"[",3)
```

11.2 Map Ensembl identifier to Affymetrix probe sets

The expression data were generated using the `Mouse_430_2` oligonucleotide microarray platform from Affymetrix. Using biomaRt, we create a mapping of Ensembl gene identifiers to the probe set identifiers on that microarray design.

```
> ensembl <- useMart("ensembl", dataset="mmusculus_gene_ensembl")
> bmRes <- getBM(attributes=c("ensembl_gene_id","affy_mouse430_2"),
+               filters="ensembl_gene_id", value=arrayGenes,
+               mart=ensembl)
> bmRes <- subset(bmRes, nchar(affy_mouse430_2)>0)
> arrayGenesToProbeSets <- split(bmRes[["affy_mouse430_2"]],
+                                bmRes[["ensembl_gene_id"]])
```

How many probe sets are mapped to each gene?

```
> table(listLen(arrayGenesToProbeSets))

   1    2    3    4    5    6    7    8    9   11   12 
6252 4625 2215  977  377  133   47   12    3    1    1 
```

Software versions

This supplement was generated using the following package versions:

- R version 2.8.0 Under development (unstable) (2008-09-13 r46541), `x86_64-unknown-linux-gnu`
- Locale: `LC_CTYPE=en_US.ISO-8859-1;LC_NUMERIC=C;LC_TIME=en_US.ISO-8859-1;LC_COLLATE=en_US.ISO-885`

- Base packages: base, datasets, graphics, grDevices, methods, splines, stats, tools, utils
- Other packages: affy 1.19.4, affyio 1.9.1, annotate 1.19.2, AnnotationDbi 1.3.9, Biobase 2.1.7, biomaRt 1.15.1, ccTutorial 0.9.5, codetools 0.2-1, DBI 0.2-4, digest 0.3.1, fortunes 1.3-5, genefilter 1.21.3, geneplotter 1.19.5, GO.db 2.2.3, graph 1.19.5, lattice 0.17-14, limma 2.15.11, preprocessCore 1.3.4, RColorBrewer 1.0-2, RCurl 0.9-4, Ringo 1.5.11, RSQLite 0.7-0, SparseM 0.78, survival 2.34-1, topGO 1.9.0, vsn 3.7.6, weaver 1.7.0, xtable 1.5-3
- Loaded via a namespace (and not attached): cluster 1.11.11, grid 2.8.0, KernSmooth 2.22-22, XML 1.96-0

Acknowledgments

This work was supported by the European Union (FP6 HeartRepair, LSHM-CT-2005-018630).

References

[1] Leisch F (2002) Dynamic generation of statistical reports using literate data analysis. In: Haerdle W, Roenz B, editors, Compstat 2002 - Proceedings in Computational Statistics. Physika Verlag, Heidelberg, Germany, pp. 575–580. 2

[2] Smyth GK (2005) Limma: linear models for microarray data. In: Gentleman R, Carey V, Huber W, Irizarry R, Dudoit S, editors, Bioinformatics and Computational Biology Solutions Using R and Bioconductor. Springer, pp. 397–420. 3

[3] Fischer JJ, Toedling J, Krueger T, Schueler M, Huber W, et al. (2008) Combinatorial effects of four histone modifications in transcription and differentiation. Genomics 91:41–51. 4, 5, 14

[4] Barrera LO, Li Z, Smith AD, Arden KC, Cavenee WK, et al. (2008) Genome-wide mapping and analysis of active promoters in mouse embryonic stem cells and adult organs. Genome Res 18:46–59. 5, 21

[5] Slater GSC, Birney E (2005) Automated generation of heuristics for biological sequence comparison. BMC Bioinformatics 6:31. 8

[6] Rennie C, Noyes HA, Kemp SJ, Hulme H, Brass A, et al. (2008) Strong position-dependent effects of sequence mismatches on signal ratios measured using long oligonucleotide microarrays. BMC Genomics 9:317. 8

[7] Durinck S, Moreau Y, Kasprzyk A, Davis S, Moor BD, et al. (2005) BioMart and Bioconductor: a powerful link between biological databases and microarray data analysis. Bioinformatics 21:3439–3440. 10

[8] Birney E, Andrews TD, Bevan P, Caccamo M, Chen Y, et al. (2004) An overview of Ensembl. Genome Res 14:925–928. 10

[9] Ashburner M, Ball CA, Blake JA, Botstein D, Butler H, et al. (2000) Gene ontology: tool for the unification of biology. The Gene Ontology Consortium. Nat Genet 25:25–29. 12

[10] Hamajima N, Matsuda K, Sakata S, Tamaki N, Sasaki M, et al. (1996) A novel gene family defined by human dihydropyrimidinase and three related proteins with differential tissue distribution. Gene 180:157–163. 14

[11] Bourgon RW (2006) Chromatin-immunoprecipitation and high-density tiling microarrays: a generative model, methods for analysis, and methodology assessment in the absence of a "gold standard". Ph.D. thesis, University of California, Berkley, Berkley, California, USA. URL `http://www.ebi.ac.uk/~bourgon/papers/bourgon_dissertation_public.pdf`. 16

[12] Liu CL, Kaplan T, Kim M, Buratowski S, Schreiber SL, et al. (2005) Single-nucleosome mapping of histone modifications in *S. cerevisiae.* PLoS Biology 3:e328. 16

[13] Kuan PF, Chun H, Keleş S (2008) CMARRT: a tool for the analysis of ChIP-chip data from tiling arrays by incorporating the correlation structure. Pac Symp Biocomput :515–526. 16
